# Cancer du sein chez l’homme: à propos de 40 cas et revue de la littérature

**DOI:** 10.11604/pamj.2017.28.287.13527

**Published:** 2017-12-04

**Authors:** Meriem Elbachiri, Safini Fatima, Zineb Bouchbika, Nadia Benchekroun, Hassan Jouhadi, Nezha Tawfiq, Souha Sahraoui, Abdellatif Benider

**Affiliations:** 1 Centre Mohamed VI pour le Traitement des Cancers, Casablanca, Maroc

**Keywords:** Homme, cancer du sein, pronostic, traitement, Man, breast cancer, prognosis, treatment

## Abstract

Le cancer du sein chez l'homme est une affection rare représentant environ 1% de tous les cancers du sein et moins de 1% de l'ensemble des néoplasies masculines. L'objectif de notre étude est de décrire les différents aspects cliniques, histologique, pronostic et thérapeutiques de cette tumeur rare afin de contribuer à l'amélioration de la prise en charge de cette affection. Il s'agit d'une étude rétrospective portant sur 40 patients colligés au Centre Mohamed VI pour le traitement des cancers de Casablanca de Janvier 2000 à Décembre 2012. L'âge médian a été de 62 ans, le délai moyen de consultation a été de 12 mois, l'autopalpation d'un nodule péri aréolaire a été le principal motif de consultation dans 90% des cas. Le carcinome canalaire infiltrant a été le type histologique prédominant dans 90% des cas. La prise en charge thérapeutique multimodale a consisté en une mastectomie suivie d'un traitement adjuvant à type de chimiothérapie, radiothérapie et/ou hormonothérapie en fonction du stade de la tumeur et des caractéristiques histologiques. Le suivi moyen a été de 38 mois, l'évolution a été caractérisée par une rémission complète chez 16 patients (soit 40%), rechute locale chez 3 patients (soit 7.5%) et par une rechute métastatique chez 5 patients (soit 12.5%). Le site métastatique le plus fréquent a été l'os (62%), suivi par le poumon et le foie. Le décès était survenu chez 10 patients (25%). Le cancer du sein chez l'homme présente des similitudes avec le cancer mammaire chez la femme. Mais il présente aussi beaucoup de particularités, d'où l'intérêt de mener des études prospectives randomisés à plus large échelle afin d'améliorer la prise en charge et le pronostic de cette affection dont l'impact psychosociale est considérable.

## Abstract (en)

Breast cancer in men is rare, accounting for approximately 1% of all breast cancers and less than 1% of all neoplasias in men. This study aimed to highlight the clinical histological, prognostic and therapeutic features of this rare tumor in order to contribute to improve the management of these patients. We conducted retrospective study of 40 patients whose data were collected at the Mohammed VI Center for Cancers Treatment in Casablanca from January 2000 to December 2012. The average age was 62 years, the mean consultation time was 12 months, self-exam of a peri-areolar nodul was the main reason for consultation in 90% of cases. Infiltrating ductal carcinoma was the predominant histological type in 90% of cases. Multimodal treatment was based on mastectomy followed by adjuvant therapy including chemotherapy, radiation therapy and/or hormonal therapy, depending on tumor stage and its histological features. The mean follow-up time was 38 months, patient's evolution was characterized by complete remission in 16 patients (40%), local recurrence in 3 patients (7.5%) and metastatic recurrence in 5 patients (12.5%). Metastases mainly occurred in the bones (62%), followed by the lungs and the liver. 10 patients (25%) died. Breast cancer in men is similar to breast cancer in women. However it has its own peculiarities, hence the importance of conducting broader prospective randomised studies in order to improve the treatment and the prognosis of this disease with a high psychosocial impact.

## Introduction

Le cancer du sein chez l'homme est une affection rare représentant environ 1% de tous les cancers du sein et moins de 1% de l'ensemble des néoplasies masculines [1]. C'est une pathologie méconnue du grand public dont le diagnostic est souvent tardif rendant le pronostic plus réservé [1, 2]. Il existe des référentiels bien codifié de prise en charge thérapeutique chez la femme ce qui n'est pas le cas chez l'homme vu le faible nombre d'études prospectives randomisées sur ce type d'affection. La prise en charge de cette affection n'est pas encore standardisée et calquée principalement sur celle de la femme. En effet, chez l'homme, cette affection est moins bien connuesur le plan biologique et thérapeutique [3]. Le traitementest pluridisciplinaire faisant appel à la chirurgie, la radiothérapie et la chimiothérapie ainsi que les thérapeutiques innovantes améliorant la survie globale des patients [4]. Notre étude est une première réalisée au Centre Mohammed VI pour le traitement des cancers dont l'objectif est de décrire et comparer les différents aspects cliniques, thérapeutiques et évolutifs du cancer du sein chez l'homme, les comparer aux données de la littérature dans le but de contribuer à l'amélioration de la prise en charge des patients souffrant de cette pathologie dont l'impact psychosociale est considérable.

## Méthodes

Il s'agit d'une étude rétrospective descriptive d'une cohorte de patients de sexe masculin traités pour cancer du sein au Centre Mohamed VI pour le traitement des cancers à Casablanca entre janvier 2000 et décembre 2012. Les critères d'inclusion ont été les patients de sexe masculin > 18ans atteints de cancer du seinlocalisé, localement avancé ou métastatiqueconfirmés histologiquement et ayant bénéficié d'une prise en charge thérapeutique dans notre structure. Les critères d'exclusion ont été les dossiers inexploitables ou sans confirmation histologiques, l'abandon du traitement. Ainsi, nous avons retenus 40 dossiers au total. L'exploitation des dossiers a été faite selon une fiche d'exploitation préétablie incluant les caractéristiques cliniques, para cliniques, thérapeutiques et évolutives. Le diagnostic du cancer du sein a été fait par biopsie de la tumeur, la stadification a été établie selon la nouvelle classification TNM. le grade histologique établie selon système histologique SBR (scarf bloom and Richardson). Une relecture des lames nous a permis de compléter l'étude immuno histochimique pour déterminer le statut des récepteurs hormonaux, le pourcentage du ki67 et l'hercept test, ce qui nous a permis de classer nos patients selon leur profil moléculaire en luminal A, luminal B, triple négatif, her2 enrichi. L'analyse statistique des données a été effectuée par SPSS dans sa version 20.Lesvaleurs de p < 0.05 ont été retenues comme significatives dans toutes les analyses.

## Résultats

**Figure 1:**
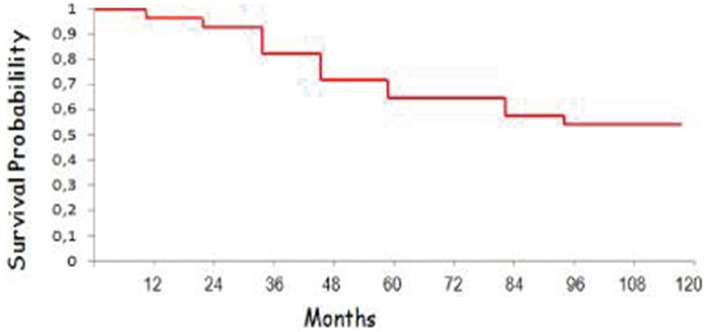
Survie globale de la cohorte

Quarante patients ont été colligés au Centre Mohamed VI pour le traitement des cancers entre Janvier 2000 et Décembre 2012. Le délai moyen entre le début de la symptomatologie et la consultation a été de 12 mois (3-28). Des antécédents familiaux de cancer du sein ont été retrouvés chez 6 patients (soit 15%). La symptomatologie a été dominée par l'autopalpation d'un nodule rétroaréolaire. Une gynécomastie a été retrouvé dans 3 cas (soit 7,5%) et la maladie de Paget a été retrouvé dans 1 cas (soit 2,5%). Les patients ont été classé selon la classification TNM et selon leur profil moléculaires, 15 patients (soit 37,5%) ont été d'emblée métastatiques (Tableau 1). Le type histologique le plus fréquent a été le carcinome canalaire infiltrant dans 91% des cas, 1 cas de carcinome colloide muqueux (soit 2,5%), 3 cas de carcinome medullaire (soit 7,5%) et 2 cas de carcinome canalaire in situ (soit 5%). L'envahissement ganglionnaire a concerné 31 patients (soit 77,5%). Par rapport à la classification histopronostique SBR, 62.5% des patients ont été classés SBR II, 30% SBRIII et 7.5% SBRI. Concernant la classification moléculaire, le profil luminal B a été prédominant avec 42.5% des cas, le profil her 2enrichie a concerné sept patients (soit 17,5%) et sept patients ont été triple négatifs (soit 17,5%). Trent et un patients (soit 82,5 %) ont bénéficié d'une mastectomie radicale modifiée (patey-madden), 2% des patients ont eu une résection musculaire partielle. Le curage ganglionnaire a été effectué dans 90% des cas. Tous les patient ont bénéficié d'un traitement adjuvant. Une radiothérapie a été délivrée chez 27patients (soit 67,5%), avec une dose de 50 Gy sur la paroi et les aires ganglionnaire. La toxicité aigue de la radiothérapie a été à type de mal de rayons dans 41% des cas et de radiodermite dans 40% des cas. Une chimiothérapie séquentielle a été administré en néo adjuvant chez 5 patients (soit 12,5%), en adjuvant chez 20 patients (soit 50%)et 15 patients d'emblée métastatiques ont eu une chimiothérapie palliative (soit 37,5%). Les principaux effets secondaires de la chimiothérapie ont été des nausées vomissements dans 99% des cas, mucite dans 70% des cas dont un grade 3 chez 4 patients, hypersensibilité au taxanes dans 75% des cas, aplasie fébrile dans 20% des cas. Une hormonothérapie type tamoxiféne a été prescrite chez tous les patients luminaux (soit 65%) exprimant les récepteurs hormonaux. Avant l'an 2008, on ne disposait pas de l'herceptine à l'hôpital, et donc l'herceptine n'a pu être administré qu'après 2008 chez les patients her2+++ ou présentant une amplification du gène her2 (Tableau 2, modalités thérapeutiques). Dans notre série, 7 patients (soit 17,5%) exprimait le gène Her2 et le traitement par trastuzumab a été administré chez 4 patients dont 1 en situation métastatique. Après un suivi médian de 38mois, L'évolution a été caractérisée par: une rémission complète chez 16 patients (soit 40%); rechute locale chez 3 patients (soit 7,5%); rechute métastatique chez 5 patients (soit 12,5%); décès chez 10 patients (soit 25%); patients ont été perdus de vue(soit 15%). Les principaux sites métastatiques ont été l'os dans 62% des cas, suivi par le poumon dans 30% des cas, le foie dans 25% des cas et la peau dans 17% des cas. La survie globale à 5ans et à 10ans a été respectivement de 62% et 51% (P < 0.05) (Figure 1).

**Tableau 1:** Tableau descriptif des caractéristiques clinicopathologiques des patients de notre série

Age	Nombre de cas	pourcentage
**18-49**	3	7,5%
**50-59**	12	30%
**60-69**	20	50%
**SYMPTOMES**		
Nodule rétroaréolaire	36	90%
Gynécomastie	3	7,5%
Maladie de paget	1	2,5%
**TAILLE TUMORALE**		
TX	2	5%
T1	4	10%
T2	5	12,5%
T3	13	32,5%
T4	16	40%
**Atteinte ganglionnaire**		
NX	3	7,5%
N0	7	17,5%
N1	15	37,5%
N2	12	30%
N3	3	7,5%
**Métastase**		
M0	25	62,5%
M1	15	37,5%
**STADE**		
I	1	2,8%
IIA	3	7,5%
IIB	5	12,5%
III	16	40%
IV	15	37,5%
**Grade**		
SBR1	3	7,5%
SBR2	25	62,5%
SBR3	12	30%
**Statut Ganglionnaire**		
PN0	9	22,5%
PN+	31	77,5%

**Tableau 2:** Modalités thérapeutique en fonction du stade TNM et du profil moléculaire

		Profil moléculaire	chirurgie	chimiothérapie	radiothérapie	hormonothérapie	herceptine
Stade I N=1		Luminal A	1cas	0	0	1cas	0
STADE II N=8	A	Lum A(n=1)	1cas	1cas	1cas	1cas	0
		Lum B(n=2)	2cas	2cas	2cas	2cas	0
		Triple nég(n=0) Her2+++(n=0)					
	B	Lum A(n=2)	2cas	2cas	1cas	2cas	0
		Lum B(n=1)	1cas	1cas	1cas	1cas	0
		Triple neg(n=1)	1Cas	1cas	1cas	0	0
		HER2+++ (n=1)	1cas	1cas	1cas	1cas	1cas
STADE III N=16		Lum A(n=4)	4cas	4cas	3cas	4cas	0
		Lum B(n=7)	7cas	7cas	7cas	7cas	0
		Triple nég(n=2)	2cas	2cas	2cas	0	0
		Her2+++(n=3)	3cas	3cas	3cas	0	2cas
STADE IV		LumA(n=1)	0	1cas	0	1cas	0
N=15		Lum B(n=7)	5cas	7cas	2cas	5cas	0
		Triple nég (n=4)	1cas	4cas	1cas	0	0
		Her2+++(n=3)	2cas	3cas	2cas	0	1cas

## Discussion

Le cancer du sein chez l'homme est une pathologie rarissime représentant dans les pays occidentaux 0,5 à 1% des cancers mammaires et 0,4 à 1,2% de tous les cancers masculins [4]. La première description remonte à 1307 et fut faite par un chirurgienanglais, John of Arderne [5]. Son incidence a connu une nette recrudescence ces 25 dernières années [6]. Au Maroc, l'incidence duancer du sein chez l'homme selon nos deux registres nationaux (Registre des cancers de Rabat et Registre des cancers de la région du grand Casablanca) est estimée à 0,8-1%. Dans les pays occidentaux, l'âge de survenuese situe approximativement entre 60 et 65 ans [6], soit environ 8 à 10 ans plustard que chez la femme [6, 7]. Dans notre série, l'âge médian est de 62 ans ce qui concorde avec la médiane d'âge des autres séries de la littérature. L'étiopathogénie reste inconnue, étant donné la rareté de cette maladie [8]. Pour de petits sous-groupes de patients, certains facteurs de risque ont pu être mis en évidence [8 , 9]. Le syndrome de Klinefelter, qui associe une trisomie XXY, un hypogonadisme avec stérilité et une gynécomastie, comporte un risque de 3 à 6% de développement de carcinome mammaire [8, 9]. L'âge moyen de développement d'un cancer du sein chez un homme ayant un syndrome de Klinefelter est de 58 ans [9]. Trois à quatre pour cent des cancers du sein chez l'homme sont associés à un syndrome de Klinefelter [9]. Les irradiations thoraciques dans l'enfance: dans une revue de Kinne, 11 cas ont été rapportés; il s'agissait de nourrissons traités pour un thymome compressif et d'adolescents porteurs de gynécomasties ou ayant eu des radioscopies répétées pour tuberculose. Quelques cas survenus après irradiation pour maladie de Hodgkin ont été également rapportés [8-10]. La cryptorchidie et L'atrophie testiculaire, quelle qu'en soit l'origine (traumatique, iatrogène, infectieuse), peuvent augmenter le risque [8-11]. Les antécédents familiaux augmentent le risque du cancer du sein comme c'est le cas chez la femme. En général, pour J.R. Weiss [12], une histoire familiale de cancer du sein chez un homme ou une femme au premier degré multiplie le risque par deux à trois. Les mutations du BRCA2 chez l'homme sont estimées de 4 à 16% [13]. D'où l'intérêt d'un conseil génétique qui doit être proposé à chaque cas. Le risque cumulatif de cancer du sein chez l'homme est de 6,3% à 70 ans [13]. L'hypothèse étiopathogénique serait celle d'un déséquilibre entre androgènes et estrogènes, comme dans certaines gynécomasties [14]. Dans notre série, un patient irradié pour maladied'hodgkin a présenté un cancer du sein 20 ans après son irradiation, notre série rapporte aussi trois cas de gynécomastie, six cas d'histoire familiale de cancer ont été rapporté mais aucun n'a eu d'enquête génétique. Le cancer du sein chez l'homme se présente, dans la plupart des cas, sous la forme d'une tuméfaction douloureuse subaréolaire, d'une rétraction mamelonnaire ou d'un écoulement sanglant [15]. Le délai entre les premiers symptômes et le diagnostic est plus tardif que chez les femmes [15]. Dans notre série, tous nos patients ont consulté pour un nodule rétro mamelonnaire. La sensibilité et la spécificité de la mammographie dans le diagnostic du cancer du sein chez l'homme sont de 90 et 92% respectivement [16]. La topographie rétroaréolaire et la faible épaisseur de tissu glandulaire expliquent la fréquence élevée de formes avancées T4 (avec fixité au pectoral et/ou ulcération cutanée), surtout dans les études anciennes [15, 16]. Dans notre série, la plupart des tumeurs ont été diagnostiqué a un stade avancé ce qui concorde avec les résultats de la littérature. La bilatéralité (synchrone et/ou métachrone) semble moins fréquente que chez la femme. Crichlow rapporte un taux global de 1, 4% [15]. La stadification repose toujours sur la classification TNM, Comme chez la femme, le bilan d'extension comprend les mêmes examens (cliché thoracique, échographie hépatique, scintigraphie osseuse et dosage du CA 15-3) [15-17].

L'homme ne possède pas d'éléments lobulaires, le type histologique le plus fréquent est le carcinome invasif ductale (IDC) (85-95%) [1-10]. Notre série rapporte des résultats similaires avec un ratio de 96%. Le cancer du sein inflammatoire et le type histologique lobulaire sont exceptionnellement décrit chez l'homme [11-16]. Par rapport au cancer du sein chez la femme, le cancer du sein chez l'homme exprime le plus souvent les récepteurs hormonaux [8, 16]. Dans notre série, 67,5% des patients expriment les récepteurs hormonaux. Une étude récente sur une série de 75 patients a montré que 5% des cancers du sein chez l'homme ont une surexpression en her2-neu ([15-17]. dans notre série 17,5% expriment le gène her2 ceci est expliqué par la faible taille d'échantillon de notre cohorte. Le cancer du sein chez l'homme semble avoir un pronostic plus péjoratif que chez la femme [14-16]. La taille tumorale ainsi que l'atteinte ganglionnaire sont deux facteurs pronostiques importants dans le cancer du sein chez l'homme [3]. Les hommes ayant une tumeur de 2 à 5cm ont un risque de décès majoré de 40% par rapport à ceux dont la tumeur mesure moins de 2cm de diamètre maximum [3-8]. Nous n'avons pas trouvé, dans la littérature, de précisions sur les stades de découverte de cancer du sein chez l'homme comparé au stade de diagnostic chez la femme. En cas d'atteinte ganglionnaire, il y a un risque supplémentaire de 50% de décès qu´en cas de ganglions indemnes de métastases [4-8]. En analyse uni variée, la négativité des récepteurs hormonaux et le grade tumoral sont associés à un mauvais pronostic de survie [3, 14]. Le cancer du sein chez l'homme dû à une mutation du BRCA2 survient plus tôt et avec un pronostic plus sombre [13, 14]. En général, le pronostic pour les patientes et les patients avec un cancer du sein est similaire [2-4, 13-15]. La stratégie thérapeutique de la prise en charge des cancers chez l'homme est similaire à celle de la femme [9, 10]. Au stade précoce, la plupart des hommes sont traités par une mastectomie radicale modifiée associée à un curage axillaire ou à la lymphadénectomie sélective (1-5;17-18). Dans une série de 31 cas de carcinome canalaire in situ, Cutuli et al. montrent trois rechutes après six tumorectomies (50%) alors qu'ils ne retrouvent qu'un seul cas de rechute pour 25 mastectomies. La petite taille de la glande mammaire rend difficile le passage en marges saines [18]. La tumorectomie n'est donc pas recommandée [12-15]. Donc, le traitement chirurgical conservateur n'a pas d'indication dans le traitement du cancer du sein chez l'homme du fait du faible volume mammaire et de l'acceptation aisée de la mastectomie par les hommes. La radiothérapie post opératoire améliore le contrôle local et la survie sans progression mais sans impact sur la survie globale [17-20]. Dans notre série, l'indication de radiothérapie a été retenue chez 27 de nos patients. L'hormonothérapie type tamoxiféne est considéré comme le standard thérapeutique en adjuvant chez les patients exprimant les récepteurs hormonaux, l'efficacité et la tolérance de ce médicament ont fait l'objet de peu d'études chez l'homme [12-16]. Les principaux effets secondaires reste le risque de complications thromboemboliques, bouffée de chaleur et diminution de la libido [18].

Dans notre série, 19 patients ont reçu une hormonothérapie adjuvante type tamoxiféne. Par analogie avec la femme, la chimiothérapie adjuvante séquentielle est indiquée chez les patients jeunes avec envahissement ganglionnaire ou éventuellement avec des lésions SBR III [15]. Il existe peu d'informations concernant l'efficacité de la chimiothérapie en adjuvant en cas de cancer du sein chez l'homme. Une seule étude prospective a été publiée dans ce but chez 24 hommes ayant bénéficié d'une chimiothérapie par CMF (cyclophosphamide, méthotrexate, fluoro-uracile) avec un taux de survie de plus de 80% à cinq ans, et significativement plus important que dans une cohorte similaire [19]. Des séries rétrospectives ont montré la diminution du risque de récurrence chez les patients [11-16]. Ce sont souvent les mêmes protocoles de chimiothérapie qui sont utilisés pour la femme. Dans le centre de l'université de Texas M.D. Anderson Cancer [17-20], la chimiothérapie est indiquée si la taille tumorale est supérieure à 1cm et en cas d'atteinte ganglionnaire. Les anthracyclines sont proposées seules si les ganglions sont indemnes et en association avec les taxanes en cas d'atteinte ganglionnaire [16-20]. Au stade métastatique: l'attitude thérapeutique est la même que chez la femme. L'hormonothérapie est souvent indiquée étant donné la positivité fréquente des récepteurs. Farrow et Adair [16] ont décrit le cas d'un cancer du sein chez l'homme ayant régressé après orchidectomie. Historiquement, l'orchidectomie, la surrénalectomie et l'hypophysectomie ont été pratiquées afin de contrôler le cancer du sein métastatique mais sont remplacées actuellement par l'hormonothérapie. Le tamoxifène est la molécule de choix avec un taux de réponse de 50% [15-18]. Les agonistes de la LH-RH ont également été utilisés avec ou sans les anti androgènes et ont prouvé leur efficacité dans le cancer du sein métastatique chez l'homme [17]. Dans notre série, 6 patients métastatiques ont reçu une hormonothérapie de première ligne type tamoxiféne. La chimiothérapie trouve sa place chez les patients ayant des récepteurs hormonaux négatifs ou bien en cas de résistance à une hormonothérapie de première ligne [17-18]. L'efficacité du trastuzumab en cas de surexpression chez l'homme n´est pas prouvée mais serait à tenter chez les hommes métastatiques ayant une surexpression en HER-2 pour Volm et al [17]. La chimiothérapie palliative en cas de progression rapide de la maladie peut être indiquée [17-18]. La survie globale à 5 et 10ans du cancer du sein chez l'homme est aux alentours de 60 et 40%.

## Conclusion

Cette étude souligne le retard diagnostic et thérapeutique de cette affection chez l'homme contrairement à celui de la femme ce qui constitue un facteur pronostic. Le nombre de ganglions envahis et la taille tumorale sont aussi de puissants facteurs pronostic, l'autre facteur pronostic péjoratif reste l'âge avancé au diagnostic surtout en cas de présence de co-morbidités ce qui pourrait limiter les choix et les possibilités thérapeutiques d'où l'intérêt de mener des études prospectives randomisés a plus large échelle afin d'améliorer la prise en charge et le pronostic de cette affection dont l'impact psychosociale est considérable.

### Etat des connaissances actuelles sur le sujet

- Tumeur rare;
- Fréquence du type canalaire exprimant les récepteurs hormonaux dans la majorité des cas;
- Traitement réalisé par analogie à celui du cancer du sein chez la femme.

### Contribution de notre étude à la connaissance

- Première étude réalisée au Centre Mohammed VI pour le traitement des cancers dont l'objectif est de décrire et comparer les différents aspects cliniques, thérapeutiques et évolutifs du cancer du sein chez l'homme;
- Notre étude a la particularité de rapporter le profil moléculaire qui constitue un élément rarement décrit dans les anciennes publications chez les hommes souffrant de cette affection.

## Conflits d’intérêts

Les auteurs ne déclarent aucun conflit d'intérêts.
